# Cytoprotective Effect of Recombinant Human Erythropoietin Produced in Transgenic Tobacco Plants

**DOI:** 10.1371/journal.pone.0076468

**Published:** 2013-10-04

**Authors:** Farooqahmed S. Kittur, Mamudou Bah, Stephanie Archer-Hartmann, Chiu-Yueh Hung, Parastoo Azadi, Mayumi Ishihara, David C. Sane, Jiahua Xie

**Affiliations:** 1 Department of Pharmaceutical Sciences, Biomanufacturing Research Institute & Technology Enterprise, North Carolina Central University, Durham, North Carolina, United States of America; 2 Complex Carbohydrate Research Center, University of Georgia, Athens, Georgia, United States of America; 3 Carilion Clinic and Virginia Tech Carilion School of Medicine, Roanoke, Virginia, United States of America; East Carolina University, United States of America

## Abstract

Asialo-erythropoietin, a desialylated form of human erythropoietin (EPO) lacking hematopoietic activity, is receiving increased attention because of its broader protective effects in preclinical models of tissue injury. However, attempts to translate its protective effects into clinical practice is hampered by unavailability of suitable expression system and its costly and limit production from expensive mammalian cell-made EPO (rhuEPO^M^) by enzymatic desialylation. In the current study, we took advantage of a plant-based expression system lacking sialylating capacity but possessing an ability to synthesize complex *N*-glycans to produce cytoprotective recombinant human asialo-rhuEPO. Transgenic tobacco plants expressing asialo-rhuEPO were generated by stably co-expressing human *EPO* and β1,4-galactosyltransferase (*GalT*) genes under the control of double CaMV 35S and glyceraldehyde-3-phosphate gene (*GapC*) promoters, respectively. Plant-produced asialo-rhuEPO (asialo-rhuEPO^P^) was purified by immunoaffinity chromatography. Detailed *N*-glycan analysis using NSI-FTMS and MS/MS revealed that asialo-rhuEPO^P^ bears paucimannosidic, high mannose-type and complex *N*-glycans. *In vitro* cytoprotection assays showed that the asialo-rhuEPO^P^ (20 U/ml) provides 2-fold better cytoprotection (44%) to neuronal-like mouse neuroblastoma cells from staurosporine-induced cell death than rhuEPO^M^ (21%). The cytoprotective effect of the asialo-rhuEPO^P^ was found to be mediated by receptor-initiated phosphorylation of Janus kinase 2 (JAK2) and suppression of caspase 3 activation. Altogether, these findings demonstrate that plants are a suitable host for producing cytoprotective rhuEPO derivative. In addition, the general advantages of plant-based expression system can be exploited to address the cost and scalability issues related to its production.

## Introduction

Erythropoietin (EPO) is a glyco-hormone consisting of 166 amino acid long polypeptide chain containing one *O*- and three *N*-glycan chains [1]. EPO is best known for its regulatory role in the production of red blood cells, and is widely used to treat anemia resulting from chronic renal failure, AIDS, rheumatoid arthritis, malignancies and many other types of anemia [1], [2]. In addition to its hematopoietic function, EPO was reported to have cytoprotective function [3]. A number of studies using mammalian cell-made recombinant human EPO (rhuEPO^M^) have revealed remarkable cytoprotective activities occurring independent of its hematopoietic activity in preclinical models of ischemic injury involving neuronal, cardiac and kidney cells [4]–[6]. Furthermore, rhuEPO^M^ was found to exhibit excellent protective effects in animal models of diabetes [7], diabetic nephropathy [8], wound healing [9] and autoimmune encephalomyelitis [10]. RhuEPO^M^ was shown to protect these tissues by acting at multiple levels, including inhibition of apoptosis [11], reduction of reactive oxygen species/glutamate [12], modulation of inflammation [4] and recruitment of stem cells [13]. Unfortunately, the therapeutic application of rhuEPO^M^ for tissue protection was tempered by the observations that EPO greatly amplifies brain injury [14], increases thrombotic events [15] and decreases survival rates [16] because of its hematopoietic activity. The cytoprotective doses of EPO are much higher than those required for stimulation of erythropoiesis and its hematopoietic activity at these high doses can stimulate mass production of red blood cells causing more damage [3]. Therefore, cytoprotective EPO derivatives lacking hematopoietic activity are highly desired.

Several strategies have been employed to develop cytoprotective EPO derivatives lacking hematopoietic activity. Short EPO peptides were designed and proved to be cytoprotective and nonhematopoietic [3], [17]. However, peptides as a drug have some limitations, such as challenging and costly synthesis, reduced *in vivo* stability, poor ability to cross physiological barriers and high conformational flexibility, which prevent their application for therapeutic purposes [18]. Furthermore, severe adverse off-target effects of peptide drugs have been reported recently, such as EPO peptide drug Peginesatide (www.fda.gov/Safety/Recalls/ucm340893.htm), which raises safety concerns about peptide-based drugs. In addition to EPO peptides, asialo-erythropoietin (asialo-rhuEPO) and carbamylated EPO prepared by enzymatic removal of sialic acid residues and carbamylation, respectively of rhuEPO were found to be nonhematopoietic but cytoprotective in animal models of stroke, sciatic nerve injury, spinal cord compression and ischemia-reperfusion kidney injury [19]–[21]. Asialo-rhuEPO in particular has been well documented to have multiple cytoprotective functions [19], [21], [22]. It can cross the blood-brain barrier and exert a neuroprotective effect in the central nervous system [19]. Despite many encouraging preclinical data, asialo-rhuEPO has found little or no use in clinical practice because of the high cost involved in its production. Currently, only small amount of asialo-rhuEPO for research is prepared by enzymatic removal of sialic acid residues from commercial rhuEPO^M^. This approach is however, not economically viable for large scale production because of the high cost and limited production of rhuEPO^M^. Functionally active rhuEPO is produced only in mammalian cells [23] whereas rhuEPO produced using the highly efficient and least expensive bacterial expression system is unstable because of the absence of *N*-glycan chains [24]. The high demand for commercial rhuEPO^M^ for anemia treatment together with limited production capacity has driven its price extremely high (∼4,000US$/mg) [25]. Thus, alternative methods to produce cytoprotective asialo-rhuEPO inexpensively are highly desired to realize its full therapeutic potential.

Plant-based expression systems that are cost-effective, free of human pathogen contamination, and easy to scale up in production have emerged as a potential alternative to current expression systems for producing therapeutic proteins [26], [27]. One aspect of using plants as a bioreactor that has not been fully taken advantage of is the absence of sialylating capacity [27], [28], which could be used to produce asialoglycoproteins. The facts that plants lack sialylating capacity, but can synthesize complex *N*-glycans similar to mammalian *N*-glycans [27]–[29] led us to hypothesize that they are an ideal bioreactor to produce asialo-rhuEPO. In the past, *EPO* has been stably [30]–[32] and transiently [33]–[35] expressed in plants to produce rhuEPO. Recently, we [36] and Parson et al. [37] stably co-expressed human *EPO* and β1,4-galactosyltransferase (*GalT*) genes in tobacco plants and moss, respectively to produce asialo-huEPO, an EPO glycoform with β1,4-galactose extended *N*-glycan chains, which has been shown to display broad cytoprotective activity [19], [21]–[22]. Despite successful expression of rhuEPO in plants [30]–[37], none of these studies investigated the cytoprotective function of plant-produced rhuEPO (rhuEPO^P^). In one study, Conley et al. [38] showed that crude extracts of plants expressing rhuEPO could protect kidney cells from injury. However, the authors did not purify rhuEPO from plant extracts to confirm its cytoprotective activity and to determine its glycosylation status. In the present study, we generated high asialo-rhuEPO expressing stable transgenic tobacco lines by co-expressing human *EPO* and *GalT* genes. *GalT* is the first glycosyltransferase in mammalian cells that initiates further branching of complex *N*-glycans [39]. Plants lack β1, 4-galactosyltransferase activity [29]. Therefore, it is necessary to introduce human GalT in plants to make complex human-like *N*-glycans. The *EPO* gene was placed under the control of a double CaMV 35S promoter (2×35S) to enhance the production level of asialo-rhuEPO, which reached 2.4 µg/g fresh leaf tissues in some high asialo-rhuEPO expressing transgenic lines. This allowed us to purify sufficient amount of asialo-rhuEPO^P^ for performing detailed *N*-glycan analysis, testing its cytoprotective effect, and studying its mechanism of action. The asialo-rhuEPO^P^ contained mainly paucimannosidic-type, high mannose-type and complex-type *N*-glycans, and it provided better protection to neuronal-like mouse neuroblastoma cells (N2A) from staurosporine (STS)-induced cell death compared with rhuEPO^M^. The cytoprotective effect of the asialo-rhuEPO^P^ was found to be mediated through JAK2 phosphorylation and inhibition of caspase 3.

## Materials and Methods

### Construction of expression vector A56, creation and characterization of transgenic plants

**Figure 1:**
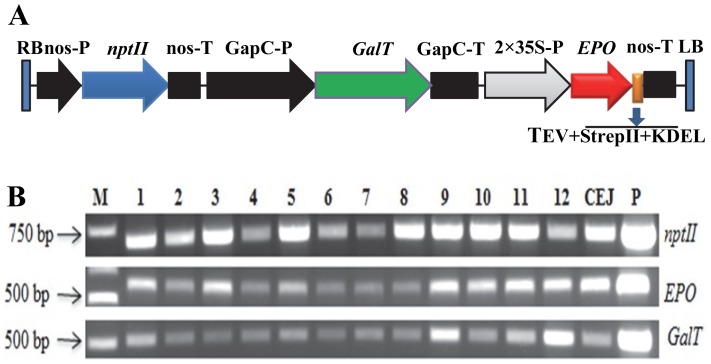
Genetic cassette used for plant transformation and evaluation of transgene integration. (A) Schematic representation of A56 genetic cassette used for tobacco plant transformation. The *EPO* coding region (red) fused with sequence encoding the TEV protease cleavage site, StrepII tag and KDEL (orange) was placed under the control of a double CaMV 35S promoter, followed by nopaline synthase terminator (nos-T). The human *GalT* coding region (green) is flanked by a glyceraldehydes-3-phosphate dehydrogease gene (*GapC*) promoter and terminator. The expression construct has a kanamycin resistance gene neomycin phosphotransferase (*nptII*) (blue) under the control of nopaline promoter (nos-P). RB/LB, right and left borders. (B) PCR analysis of A56 transgenic lines (lanes 1-12, A56-1 to -12) and CEJ120-12 (CEJ) for the presence of *EPO*, *GalT* and *nptII* in the plant genomic DNA. M, marker; P, plasmid.

A binary vector designated as A56 (**Figure 1A**) containing a 2×35S promoter and a tobacco glyceraldehyde-3-phosphate dehydrogenase gene (*GapC*) promoter (1291 bp) [40] driving human *EPO* and *GalT* [41], respectively was constructed as follows. The *Gap*C promoter was shown to direct *GUS* gene expression at high levels both in leaves and roots [40]. First, *EPO* cDNA was synthesized (Eurofins MWG Operon, Huntsville, AL, USA) with adding a *Spe*l site at 5′ end and additional 66 bp sequence at the 3′ end. This 66 bp sequence was designed to code for a TEV protease cutting site ENLYFQG, StrepII tag, an ER retention signal KDEL, and a *Sac*I site. The 2×35S promoter was created by PCR amplification using pBI121 as DNA templates based on information from an expression vector pKYLX71:35S^2^ [42]. For cloning purpose, two restriction cutting sites *Hind*III at the 5′ end and a *Spe*I site at the 3′ end were also added. The two single copies of amplified CaMV 35S were sub-cloned together into pCR®2.1 vector (Life Technologies – Invitrogen, Grand Island, NY, USA) to create a new plasmid DNA called CEJ887. The synthesized *EPO* fragment was isolated by digestion with restriction enzymes *Spe*I and *EcoR*I and sub-cloned into CEJ887 at *Spe*I and *EcoR*I sites. Resultant plasmid DNA was designated as CEJ890, which contained a 2×35S promoter driving *EPO*. Then 2×35S promoter driving *EPO* fragment was isolated from CEJ890 by digestion with *Hind*III and *Sac*I and sub-cloned into *Hind*III and *Sac*I sites of an expression vector pBI121 to replace CaMV 35S promoter and *GUS* gene. The resultant plasmid DNA (CEJ893) contained a nopaline synthase gene (*nos*) promoter driving a neomycin phosphotransferase gene (*nptII*) with a *nos* terminator and a 2×35S promoter driving *EPO* with a *nos* terminator. The *GapC* promoter driving *GalT* with *GapC* terminator was isolated from plasmid DNA CEJ120 [36] and cloned into CEJ893 by *Hind*III digestion to yield A56 (**Figure 1A**). A56 was transformed into tobacco leaf using *Agrobacterium*-mediated transformation [43]. The presence of *nptII*, *GalT* and *EPO* genes in transgenic plants was confirmed by PCR analysis as described by Kittur et al. [36].

### Extraction, quantification and purification of asialo-rhuEPO^P^

To detect the total (extractable and unextractable) asialo-rhuEPO in transgenic plants, leaf tissues (1 g) were frozen in liquid nitrogen and grounded into a fine powder using mortar and pestle. About 50 mg of powdered tissue was directly extracted with 200 µl of 4X SDS sample buffer at 95°C for 15 min. Then total leaf protein extracts (16 and 32 µl) were used for Western blot analysis along with 3, 6 and 12 ng of rhuEPO^M^ to generate a standard curve. The expression level of total asialo-rhuEPO in transgenic line was quantified using densitometry. The measurement was repeated twice. Soluble protein extracts for protein purification and other analyses were prepared as described previously [36]. The levels of asialo-rhuEPO^P^ in total soluble protein extracts were determined using a sandwich ELISA [36]. To purify asialo-rhuEPO^P^, our previous purification procedure [36] was modified by including preliminary fractionation with ammonium sulfate to remove plant pigments. Briefly, solid ammonium sulfate was added to plant extract to 25% saturation. Following centrifugation at 15,000 x *g* for 15 min, the supernatant was collected and ammonium sulfate concentration was adjusted to 65% to precipitate the asialo-rhuEPO^P^. The pellet was then dissolved in PBS and asialo-rhuEPO^P^ was purified using immunoaffinity chromatography as described previously [36]. Purified protein was stored at −20°C for future use.

### In vitro cytoprotection assay

Mouse neuroblastoma cell line (N2A) (American Type Culture Collection, Manassas, VA, USA) was maintained in Dulbecco's modified Eagle's medium (DMEM) (Thermo Scientific, Rockford, IL, USA) with high glucose, containing 10% FBS and penicillin/streptomycin (100 U/ml and 100 µg/ml, respectively) at 37°Cand 5% CO_2_. For EPO-mediated cytoprotection assay, N2A cells were seeded at a density of 4.0×10^4^ in 96-well cell culture plates for lactate dehydrogenase (LDH) assay or at a density of 8.0×10^5^ in T-25 flasks for Western blot analysis. They were incubated at 37°C in 5% CO_2_ until they reached 70% confluence. Cells were then treated simultaneously with 20 U/ml purified asialo-rhuEPO^P^ or rhuEPO^M^ (R&D Systems, Minneapolis, MN, USA) in PBS containing 0.1% BSA and 1 µM STS directly added to the medium. As a vehicle control, same volume of PBS containing 0.1% BSA was added to the medium. For STS alone treatment, 1 µM STS in PBS containing 0.1% BSA was included in the medium. Concentration of asialo-rhuEPO in purified fractions was determined using sandwich ELISA as described above, and the number of asialo-rhuEPO^P^ units was calculated from asialo-rhuEPO concentration as described by Erbayraktar et al. [19]. After 12 h of treatment, cell death was assessed with the non-radioactive cytotoxicity assay kit (Roche, Indianapolis, IN, USA) according to the manufacturer's protocol. Each test was performed using six replicates and the average of six replicates was used in the final calculations to compute cytotoxicity.

### SDS-PAGE and Western blot analysis

SDS-PAGE was carried out according to method of Laemmli [44]. For asialo-rhuEPO^P^ Western blot analysis, same protocol and antibodies were used as described previously [36]. For analyzing p-JAK2/JAK2 and caspase 3, N2A cell lysates were prepared by extracting untreated and treated cells using cytosolic protein extraction buffer [45]. NP-40 was added immediately before centrifugation at 5,000 x *g*. The supernatant was used as cytosolic protein fraction for detection of p-JAK2/JAK2 and caspase 3 by Western blot analysis. Protein separation and transfer were performed as described previously [36]. The membranes were then blocked for 1 h with 5% BSA in TBST. Blots were incubated separately with anti-caspase 3, anti-p-JAK2 (Cell Signaling, Danvers, MA, USA), anti-JAK2 (Santa Cruz Biotechnology, Dallas, TX, USA) and anti-β-actin (Sigma Chemical Company, Saint Louis, MO, USA) antibodies each at a dilution of 1∶1000 except anti-JAK2 (1∶400). Blots were incubated overnight at 4°C. Following incubation, blots were washed and incubated with 1∶1000 diluted HRP-conjugated secondary antibody for 1 h at room temperature. SuperSignal ®West Pico Chemiluminescent substrate was used to detect protein bands.

### Asialo-rhuEPO^P^ identification by LC-MS/MS

For protein identification by LC-MS/MS, about 7 µg of purified asialo-rhuEPO^P^ was separated on a 12.5% SDS/PAGE gel. The gel was fixed and stained with coomassie stain. Protein bands corresponding to immunoreactive bands 30 and 28 kD on Western blot were excised and analyzed by LC-MS/MS at Duke University proteomic facility.

### Analysis of asialo-rhuEPO^P^ *N*-glycans by nanospray ionization FTMS

For *N*-glycan analysis, about 20 µg of purified asialo-rhuEPO^P^ was separated on a 10% Mini-Protean TGX gels (BioRad, Hercules, CA, USA). After staining with coomassie stain, EPO bands were excised, and in-gel trypsin digestion was performed as described previously [46]. Glycopeptides were extracted, followed by C18 purification, PNGase A release of *N*-glycans, and C18 purification of the released glycans. Released *N*-linked oligosaccharides were dissolved in dimethylsulfoxide and then methylated with NaOH and methyl iodide. Nanospray Ionization FTMS (NSI-MS^n^) analysis was performed using a LTQ Orbitrap XL mass spectrometer (Thermo Fisher, Rockford, IL, USA) equipped with a nanospray ion source. Permethylated *N*-linked glycans were dissolved in 1 mM NaOH in 50% methanol then infused directly into the instrument at a constant flow rate of 0.5 µl/min. A full FTMS spectrum was collected at 30,000 resolutions with 3 microscans. The capillary temperature was set at 210°Cand MS analysis was performed in the positive ion mode. For total ion mapping (automated MS/MS analysis), m/z range 200 to 2,000 was scanned with ITMS mode in successive 2 mass unit windows. Binding of asialo-rhuEPO^P^ to ECA-agarose column to detect the presence of β1,4-galctose residues was performed as described previously [36].

### Statistics

ANOVA program (http://www.physics.csbsju.edu/stats/anova.html) was used for statistical analysis to compare the toxicities between treatments: STS vs STS+rhuEPO^M^, STS vs STS+asialo-rhuEPO^P^, and STS+rhuEPO^M^ vs STS+asialo-rhuEPO^P^. Levels of statistical significance were set at *P*<0.05.

## Results

### Generation of transgenic tobacco plants expressing asialo-rhuEPO

In our previous study, co-expression of *EPO* with *GalT* under the control of CaMV 35S and GapC promoters, respectively in tobacco plants resulted in low level of asialo-rhuEPO (5 ng/mg of total soluble protein, TSP) [36]. To improve the production level, a new construct A56 was created containing *EPO* driven by a strong 2×35S promoter (**Figure 1A**). The *EPO* cDNA was also fused with the TEV protease cutting site ENLYFQG, StrepII tag, and an endoplasmic reticulum (ER) retention signal KDEL at its 3′ end (**Figure 1A**). A56 was stably transferred into tobacco plants using *Agrobacterium*-mediated transformation. Of 12 putative transformants regenerated on kanamycin-medium, all showed integration of *EPO* and *GalT* genes in their genomes (**Figure 1B**).

### Quantification of asialo-rhuEPO expression levels in transgenic plants

**Figure 2:**
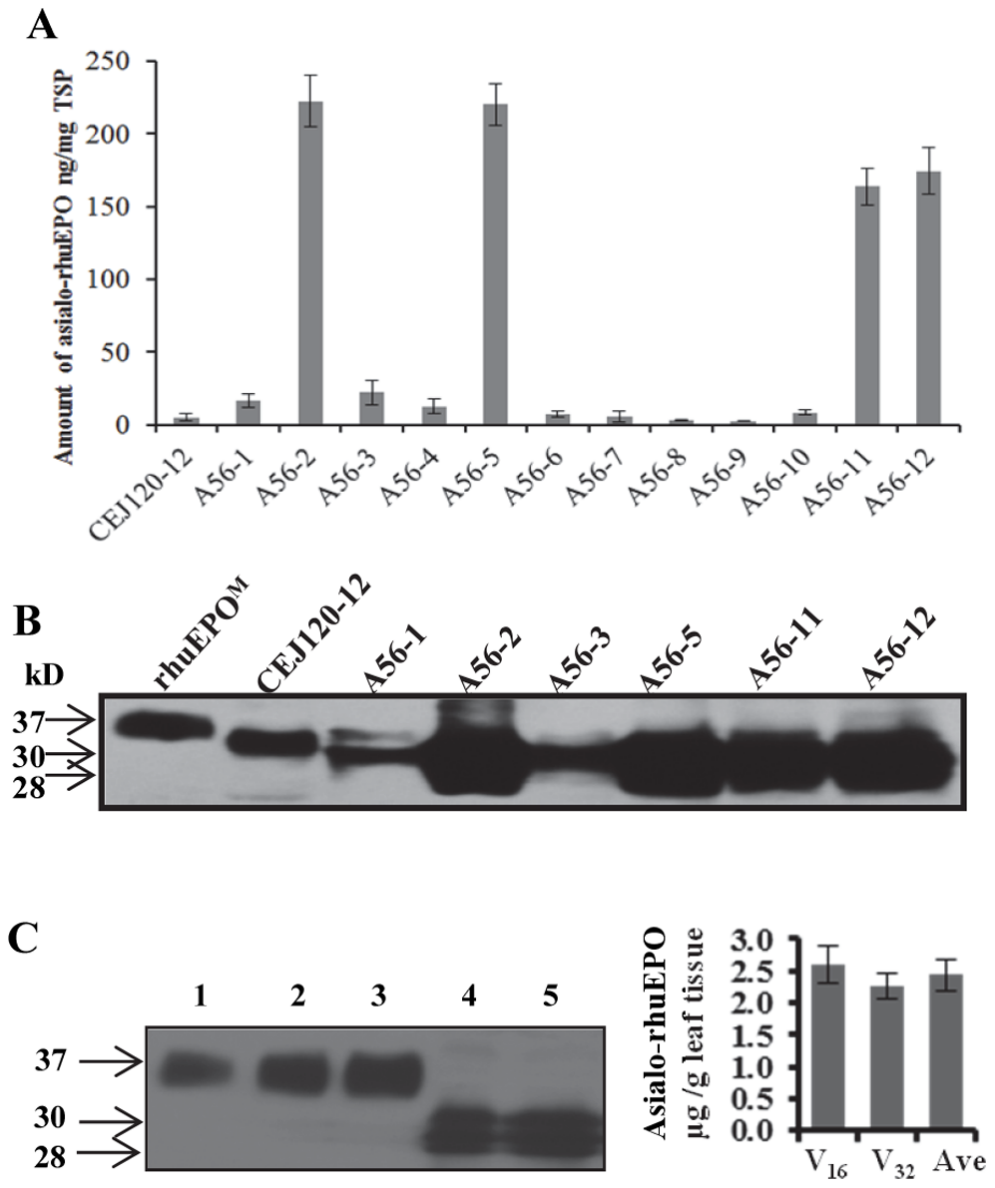
Quantification of asialo-rhuEPO in leaf extracts of transgenic tobacco plants. (A) A sandwich ELISA was used to determine accumulation levels of asialo-rhuEPO in transgenic plants expressing EPO under the control of CaMV 35S (CEJ120-12) promoter and a 2×35S (A56 1–12) promoter. All data plotted are the average of three independent measurements ± SD. (B) Western blot of total soluble protein (TSP) from CEJ120-12 and selected A56 transgenic lines. (C) Western blot of total leaf protein extracts (TLPE) isolated from transgenic line A56-5. Lanes 1 to 3, 3, 6 and 12 ng of rhuEPO^M^; lanes 4 and 5, 16 µl (V_16_) and 32 µl (V_32_), respectively of A56-5 TLPE. The arrows mark the position of rhuEPO^M^ (lane 1) and asialo-rhuEPO^P^ glycoforms. The expression level of asialo-rhuEPO in leaf tissue was calculated from standard curve generated by measuring the band intensities of known amounts of rhuEPO^M^ and amount of TLPE used. The Western blot analysis was repeated twice. All data plotted are the average of two independent measurements ± SD. Ave, average.

The asialo-rhuEPO levels in A56 transgenic tobacco lines along with our previously generated highest expression line CEJ120-12 (expressing *EPO* driven by CaMV 35S promoter) [36] were analyzed by sandwich ELISA [38]. Results showed that 10 out of 12 A56 transgenic lines expressed asialo-rhuEPO, but at different levels (**Figure 2A**). Four lines (A56-2, -5, -11 and -12) had 30- to 40-fold higher asialo-rhuEPO accumulation levels (171–231 ng/mg TSP) than the CEJ120-12 line (5 ng/mg TSP) (**Figure 2A**). Western blot analysis of soluble leaf protein extracts of four high (A56-2, A56-5, A56-11 and A56-12) and two medium (A56-1 and A56-3) asialo-rhuEPO accumulating lines along with CEJ120-12 revealed two closely migrating protein bands of sizes 28 and 30 kD when the blot was probed with anti-EPO antibody (**Figure 2B**). Asialo-rhuEPO^P^ was smaller in size than the rhuEPO^M^ (**Figure 2B**, lane 1). The predicted molecular weight of EPO with a C-terminal 22 amino acid long fusion peptide (containing TEV protease cleavage site, StrepII tag and a KDEL sequence) lacking glycan chains is 18.8 kD. The 30 and 28 kD bands are EPO glycoforms bearing *N-*glycan chains differing in length/size. The intensities of asialo-rhuEPO^P^ bands observed in these selected lines are in line with their accumulation levels measured by ELISA. To detect the total (extractable and unextractable) asialo-rhuEPO in transgenic plants, total leaf protein extracts from the highest expression line A56-5 was analyzed by Western blot. As can be seen from **Figure 2C**, two immunoreactive bands were present on the Western blot, consistent with the same number of immunoreactive bands observed in TSP (**Figure 2B**). The expression level of total asialo-rhuEPO in A56-5 line was quantified using densitometry with known amount of rhuEPO^M^ (**Figure 2C**) to generate a calibration curve. The asialo-rhuEPO level was about 2.4 µg/g of fresh leaf tissue.

### Purification, SDS-PAGE and LC-MS/MS analysis of asialo-rhuEPO^P^

**Figure 3:**
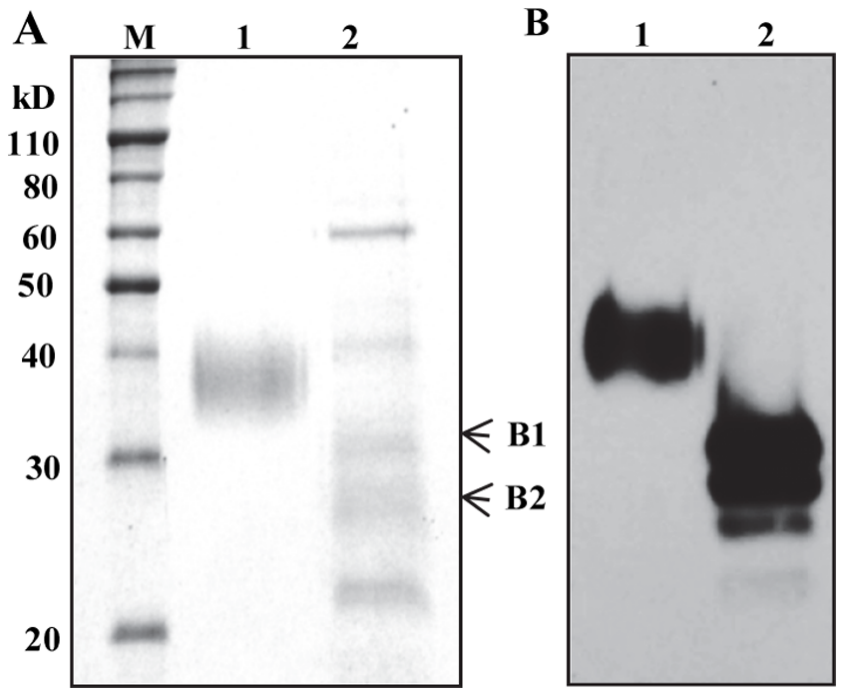
SDS-PAGE profile (*A*) and Western blot (*B*) of purified asialo-rhuEPO^P^. (A) Coomassie-stained gel showing commercial rhuEPO^M^ (lane 1, 5 µg) and the asialo-rhuEPO^P^ purified from transgenic line A56-6 (lane 2, 7 µg). M, protein markers. The arrowheads mark the protein bands, which were excised for LC-MS/MS analysis. (B) Western blot of rhuEPO^M^ (lane 1, 5 ng) and purified asialo-rhuEPO (lane 2, 15 ng).

To purify asialo-rhuEPO^P^, leaf tissues from transgenic line A56-5 grown in greenhouse were used. Initial attempts to purify strepII-tagged asialo-rhuEPO^P^ using Strep-Tactin column were unsuccessful, which might be due to inaccessibility of strepII tag to Strep-Tactin as also reported by Jez et al. [47]. To purify asialo-rhuEPO^P^, we first performed preliminary fractionation with ammonium sulfate and then followed by immunoaffinity chromatography as described previously [36]. SDS-PAGE analysis of purified asialo-rhuEPO^P^ showed five bands of sizes 66, 40, 30, 28 and 22 kD (**Figure 3A**). Western blot analysis of purified asialo-rhuEPO^P^ revealed two major and one minor immunoreactive bands corresponding to 30, 28 and 22 kD (**Figure 3B**). The 66 kD band corresponds to BSA, which was added during column binding. Since the 40 kD did not cross-react with anti-EPO antibody, it could be either a tobacco protein or breakdown product of BSA.

To further confirm whether the two major bands of sizes 30 (band 1) and 28 kD (band 2) (**Figure 3A**) correspond to rhuEPO, they were subjected to LC-MS/MS analysis. Six unique peptides, VNFYAWK, SLTTLLR, EAISPPDAASAAPLR, TITADTFR, VYSNFLR and LYTGEACR corresponding to 46–52, 104–110, 117–131, 132–139, 144–150 and 155–162 position in the mature human EPO protein sequence, could be identified from the MS/MS spectra of tryptic peptides of 30 kD protein band (**Figure S1A-G**). In the case of 28 kD band, seven unique peptides, LICDSR, VNFYAWK, MEVGQQAVEVWQGLALLSEAVLR, EAISPPDAASAAPLR, TITADTFRK, VYSNFLR and LYTGEACR representing amino acids 5–10, 46–52, 54–76, 117–131, 132–140, 144–150 and 155–162 in the mature human EPO sequence could be identified (**Figure S2A-H**). The above results indicate that both 30 and 28 kD protein bands are rhuEPO protein.

### Analysis of asialo-rhuEPO^P^ *N*-glycans

**Figure 4:**
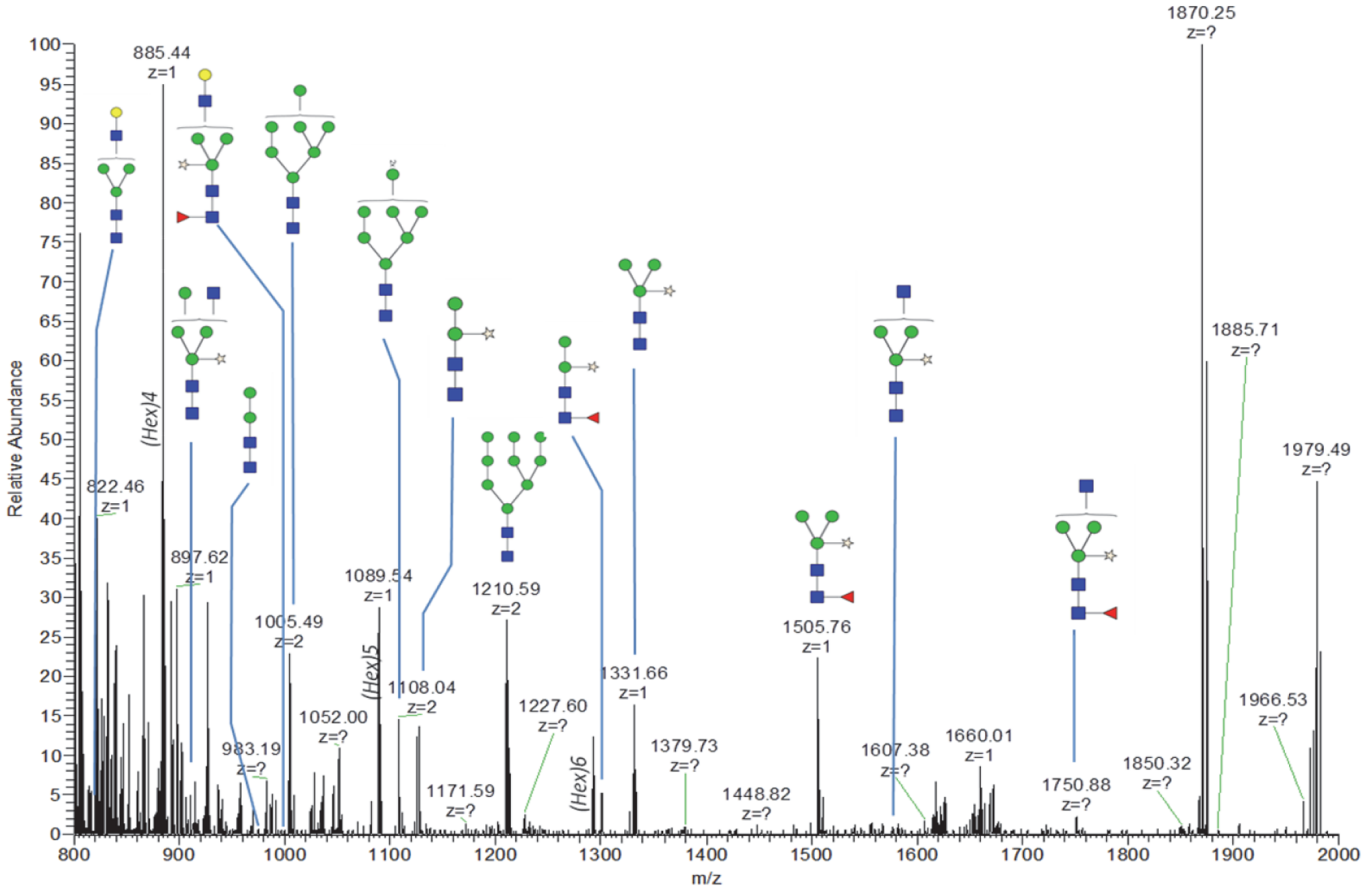
NSI-FTMS spectrum of PNGase A released and permethylated asialo-rhuEPO^P^ *N*-glycans. The schematic glycan structures of the glycans found in *N*-glycan pool of asialo-rhuEPO^P^ are shown. The structure for each peak was further verified by MS/MS analysis using total ion mapping. The symbols for the glycan structures are: filled blue square, GlcNAc; filled green circle, mannose; filled yellow circle, galactose; filled red triangle, fucose, unfilled star, xylose.

To examine the *N*-glycans of asialo-rhuEPO^P^, they were first released with the PNGase A, then purified and permethylated prior to mass spectrometric analysis. NSI-FTMS analysis of *N*-glycans of asialo-rhuEPO^P^ revealed complex mixture of at least 13 *N*-glycans (**Figure 4** **;** **Table 1**). Structures of these *N*-glycans were further confirmed by fragmentation (MS/MS) analysis using total ion mapping (**Figure S3**). The major proportion of *N*-glycans consisted of paucimannosidic-type structures (45%) (*m/z* = 1331.6, 1301.6 and 1505.7), which are typically present on plant glycoproteins. The next most abundant *N*-glycans were of high-mannose type (*m/z* = 1005.4, 1108 and 1210.5) corresponding to 33% of the total relative peak intensity. Approximately 13% of asialo-rhuEPO^P^ *N*-glycans were complex/hybrid type (*m/z* = 821.9, 988.9, 1576.8, 1750.8 and 1780.9). The molecular ions observed at *m/z* = 821.9 (*z* = 2) and 988.9 (*z* = 2) correspond to galactosylated *N*-glycans, which together represented about 4% of total *N*-glycans. In addition to these, we detected unusually high proportion (7.4%) of hitherto unreported *N*-glycan [(Xyl)Man_2_GlcNAc_2_, *m/z* = 1127.5] (**Table 1**). No sialic acid residues were detected, consistent with earlier reports of lack of sialylating capacity in plants [27]–[29].

**Table 1:** *N*-Glycans of asialo-rhuEPO^P^ identified by NSI-FTMS and MS/MS analyses.

Observed m/z	Glycan structure	Relative intensity (%)*	MS/MS results^□^
967.3 (z = 1)	Man_2_GlcNAc_2_	0.4	-HexNAc
1005.4 (z = 2)^‡^	Man_7_GlcNAc_2_	12.1	-reducing end HexNAc
1108.0 (z = 2)^‡^	Man_8_GlcNAc_2_	7.5	-reducing end HexNAc
1127.5 (z = 1)	(Xyl)Man_2_GlcNAc_2_	7.4	-Xyl, -reducing end HexNAc
1210.5 (z = 2)^‡^	Man_9_GlcNAc_2_	13.9	-reducing end HexNAc
1301.6 (z = 1)	(Xyl)Man_2_(Fuc)GlcNAc_2_	5.4	-Fuc, -Xyl
1331.6 (z = 1)	(Xyl)Man_3_GlcNAc_2_	16.8	-Xyl, -reducing end HexNAc
1505.7 (z = 1)	(Xyl)Man_3_(Fuc)GlcNAc_2_	22.9	-Xyl, -Fuc, -reducing end HexNAc
1576.8 (z = 1)	GlcNAc(Xyl)Man_3_GlcNAc_2_	0.7	-Xyl, -reducing end HexNAc
1620.8 (z = 1) 821.9 (z = 2)^‡^	GalGlcNAcMan_3_GlcNAc_2_	1.0	-Hex/HexNAc, -reducing end HexNAc
1750.8 (z = 1)	GlcNAc(Xyl)Man_3_(Fuc)GlcNAc_2_	2.3	-Xyl, -Fuc, -reducing end HexNAc
1780.8 (z = 1) 901.9 (z = 2)^‡^	GlcNAc(Xyl)Man_4_GlcNAc_2_	6.6	-Xyl, -reducing end HexNAc, -non-reducing end HexNAc
1954.9 (z = 1) 988.9 (z = 2)^‡^	GalGlcNAc(Xyl)Man_3_(Fuc)GlcNAc_2_	2.8	-Xyl, -Fuc, -HexNAc/Fuc, -HexNAc

* Percent peak intensities were calculated by pulling the intensities of each peak and calculating percentages over their total intensity. ^□^MS/MS spectra were obtained using total ion mapping. -, loss. ^‡^z = 2 refers to the doubly charged m/z species observed by NSI-FTMS. When both singly and doubly charged species were observed, both values were presented.

**Figure 5:**
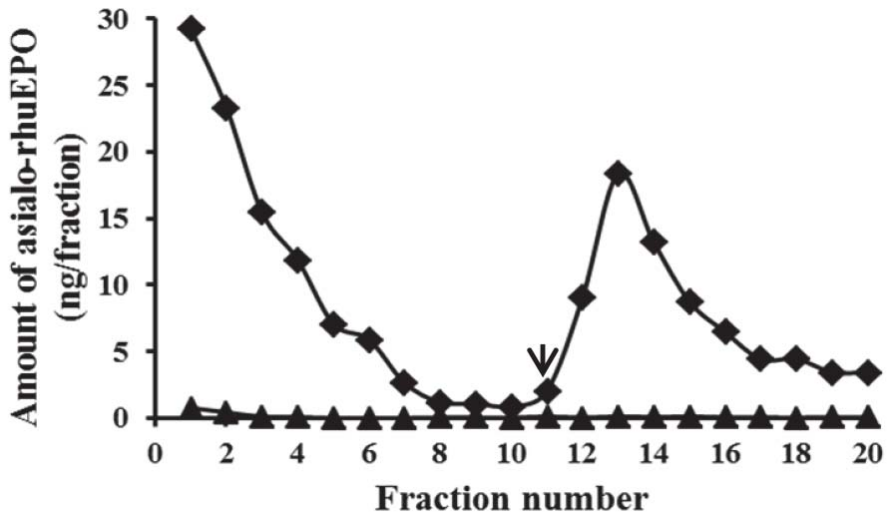
Binding profile of asialo-rhuEPO^P^ to ECA-agarose column. About 800-rhuEPO^P^ (-♦-) or plant-produced rhuEPO lacking both sialic acid and β1,4-galactose (-▴-, negative control) in HEPES-KOH buffer was applied to 1 ml ECA-agarose column. Bound protein was eluted with HEPES-KOH buffer containing 0.2 M lactose. A sandwich ELISA was used to determine the amount of rhuEPO in flow through, wash and eluted fractions. The arrow indicates the start of elution.

To investigate whether the galactose present on the asialo-rhuEPO^P^ *N*-glycans (*m/z* = 821.9 and 988.9) is β1,3- or β1,4-linked, binding of purified asialo-rhuEPO^P^ to a β1,4-galactose-specific *Erythrina cristagalli* agglutinin column (ECA–agarose) was performed. ECA exclusively binds glycan chains containing terminal β1,4-, but not β1,3-linked galactose residues [48]. As can be seen from **Figure 5**, asialo-rhuEPO^P^ readily bound to the ECA-agarose column and could be eluted with 0.2 M lactose. RhuEPO produced by expressing *EPO* alone in tobacco plants [32] didn’t bind to it (**Figure 5**). These results indicate that asialo-rhuEPO^P^ molecules indeed carry β1,4-linked terminal galactose residues, which is expected because human *GalT* was co-expressed with the *EPO*. About 9% of the asialo-rhuEPO^P^ molecules were estimated to carry β1,4-linked galactose residues based on the amount of asialo-rhuEPO^P^ applied and the amount bound to the ECA-agarose column, which is higher than that observed by NSI-FTMS analysis.

### Asialo-rhuEPO^P^ protects neuronal-like cells against STS-induced cell death

**Figure 6:**
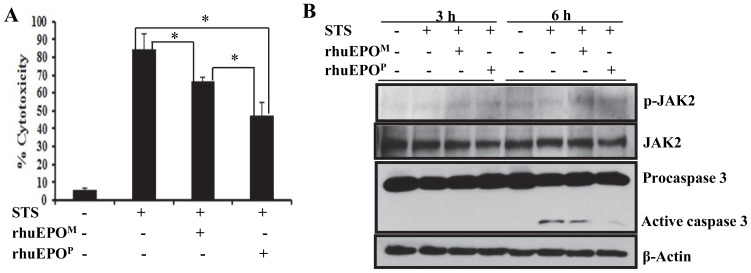
The cytoprotective effect of asialo-rhuEPO^P^ and Western blot of JAK2 and caspase 3. N2A cells were treated individually with PBS containing 0.1% BSA (vehicle control), 1 µM STS, 1 µM STS+20 U/ml asialo-rhuEPO^P^ (rhuEPO^P^) or 1 µM STS+20 U/ml rhuEPO^M^. (A) Cytotoxicity was measured by LDH assay after 12 h treatment. Each experiment had six replicates. All data plotted are the average of three independent experiments ± SD. *, *P*<0.05. (B) Western blot of activated JAK2 and caspase 3 in cell lysates prepared from cells treated by STS and rhuEPO for 3 and 6 h. For detection of p-JAK2 and JAK2, the blot was probed with anti-p-JAK antibody first followed by stripping the blot and re-probing with anti-total JAK2 antibody. Active caspase 3 was detected using an anti-caspase 3 antibody, which also cross-reacts with procaspase 3. β-actin was used as internal control.

To study its cytoprotective effect, immunoaffinity purified asialo-rhuEPO^P^ was used to study its ability to protect neuronal-like cells against STS-induced apoptosis. N2A cells were simultaneously treated with 1 µM STS and 20 U/ml asialo-rhuEPO^P^ or rhuEPO^M^ (a positive control) for 12 h. Cytotoxicity was measured by the amount of lactate dehydrogenase released into culture supernatant. Treatment of N2A cells with 1 µM STS alone resulted in 84% cytotoxicity (**Figure 6A**), whereas only 47% cytotoxicity was observed in cells simultaneously treated with STS and asialo-rhuEPO^P^ corresponding to 44% cytoprotection (**Figure 6A**). In the case of rhuEPO^M^, cytotoxicity was reduced to 66%, which corresponds to 21% cytoprotection (**Figure 6A**). The above results indicate that the asialo-rhuEPO^P^ is not only functionally active but also has even better cytoprotective effect (∼2 fold) than rhuEPO^M^.

### Asialo-rhuEPO^P^-mediated cytoprotection is via JAK2 phosphorylation and caspase 3 inhibition

JAK2 is a tyrosine kinase associated with EPO receptor (EPOR), and its phosphorylation is believed to be essential for cell survival [11], [49]. Phosphorylation of JAK2 was investigated by immunoblotting of cell lysates prepared from untreated, STS-treated and STS+asialo-rhuEPO^P^ or STS+rhuEPO^M^ treated cells for 3 and 6 h. As can be seen from **Figure 6B**, a phosphorylated JAK2 (p-JAK2) band with higher intensity was observed in cell lysates prepared from cells treated for 3 h with STS+rhuEPO^M^ and STS+asialo-rhuEPO^P^ than untreated and STS-treated cell lysates. After 6 h treatment, the p-JAK2 band intensity in STS+asialo-rhuEPO^P^ or STS+rhuEPO^M^ treated samples remained higher than STS-treated and untreated cells. When blot was stripped and re-probed with anti-JAK2 antibody, the intensity of total JAK2 band was found to be similar in all samples, indicating that JAK2 is activated by both asialo-rhuEPO^P^ and rhuEPO^M^ treatments. These results imply that cytoprotective effect of asialo-rhuEPO^P^ is mediated via phosphorylation of JAK2.

STS is a well-known inducer of apoptosis in many cell types, and caspase 3 is believed to be responsible for STS-induced apoptosis [50]. To investigate whether EPO treatment prevents STS-induced caspase 3 activation, we performed Western blot analysis on cell lysates that were prepared for JAK2 analysis. When cell lysates from 3 h treated cells were analyzed, no active caspase 3 fragment (19 kD) was detected in either sample (**Figure 6B**). After 6 h treatment, the 19 kD fragment was clearly visible in STS treated and STS+rhuEPO^M^ treated samples, but the band intensity in the latter case was much lower than that in the former one. In the case of STS+asialo-rhuEPO^P^ treated sample, a 19 kD active caspase 3 band was visible, but its intensity was significantly lower compared to those in STS treated and STS+rhuEPO^M^ treated samples (**Figure 6B**). The active caspase 3 band intensities in these samples are consistent with their cytotoxicities observed, which were 84%, 66% and 47% in STS alone, STS+rhuEPO^M^ and STS+asialo-rhuEPO^P^ treated cells, respectively. These results indicate that asialo-rhuEPO^P^ protects N2A cells by inhibiting caspase 3 activation via JAK2 phosphorylation.

## Discussion

Asialo-rhuEPO with its broader cytoprotective effects has many potential therapeutic applications. However, no expression system is available to express this glycoprotein directly, and the available method of preparation from rhuEPO^M^ by enzymatic removal of sialic acid residues is not cost-effective, which limits its applications. Plants have the capacity to synthesize complex *N*-glycans, but lack sialylating capacity [27], [51]. In this study, we exploited the lack of sialylating capacity in plants to express asialoglycoprotein by producing a cytoprotective asialo-rhuEPO^P^. Our results show that (*i*) the asialo-rhuEPO^P^ has better cytoprotective effect than rhuEPO^M^; (*ii*) the cytoprotective mechanism of asialo-rhuEPO^P^ involves inhibition of caspase 3 via receptor-initiated JAK2 phosphorylation; and (*iii*) generation of asialo-rhuEPO^P^ in plants provides a promising platform that can address the potential cost as well as scalability issues associated with large scale production.

To ensure high expression level in tobacco plants, *EPO* was expressed under the control of a strong 2×35S promoter. The level of asialo-rhuEPO in transgenic lines A56-2 and A56-5 approached 230 ng/mg TSP compared to 5 ng/mg TSP in the highest accumulating line CEJ120-12 expressing *EPO* under the control of a single copy CaMV 35S promoter [36] as confirmed by ELISA and Western blot analyses (**Figure 2A and B**). The total asialo-rhuEPO level was about 2.4 µg/g of leaf tissue in the highest expressing line A56-5. The 40-fold increase in the accumulation of asialo-rhuEPO might be due to the use of a strong 2×35S promoter [52] as well enhanced protein stability by the presence of extra amino acid sequences (TEV cleavage site and the strepII tag) at the C-terminus as reported [38], [53]. These transgenic plants with improved expression allowed us to purify asialo-rhuEPO^P^ for downstream characterization. Asialo-rhuEPO^P^ is smaller in size than rhuEPO^M^ (**Figures 2B** **and** **3B**) because it bears only smaller biantennary *N*-glycans in contrast to rhuEPO^M^, which carries larger sialylated tri-, and tetraantennary *N*-glycans [54]. Asialo-rhuEPO^P^ accumulates as two major protein bands as evident from two immunoreactive bands observed on Western blots of crude extracts (**Figure 2B**) and purified asialo-rhuEPO^P^ (**Figure 3A**), and also confirmed by LC-MS/MS analysis (see **Figures S1 A-F and S2 A-G**). These two bands may correspond to more than two glycoforms since a total of 13 *N*-glycan chains varying in sizes were identified using mass spectrometry (**Figure 4** **;** **Table 1**).

The asialo-rhuEPO^P^ bears heterogeneous *N*-glycans as evident from identification of high mannose, paucimannosidic-type and complex/hybrid *N*-glycans (**Figure 4** **;** **Table 1**). Identification of paucimannosidic-type and complex and hybrid *N*-glycans suggests that majority of the asialo-rhuEPO^P^ was passed further along the secretory pathway and retrieved back to the ER because of the presence of KDEL signal peptide. Such as retrieval mechanism has been described for plant ER resident chaperone calreticulin whose *N*-glycan chains bear xylose and fucose residues, which are typically added in the *cis* and *trans* Golgi compartments, respectively [55]. The presence of only 4-9% β1,4-galactose-extended *N*-glycans (**Figures 4** **and** **5** **;** **Table 1**) indicates that the co-expressed human GalT is functionally active, but the galactosylation efficiency is poor. One reason for the lower proportion of galactose-extended *N*-glycans could be due to processing of complex *N*-glycans by hexosaminidases (by eliminating terminal GlcNAc residues) to paucimannosidic-type structures [56]. This is supported by the fact that about 45% of the asialo-rhuEPO^P^ *N*-glycans were of paucimannosidic-type (**Table 1**). Terminal GlcNAc residues are pre-requisite for the extension of glycan chains with β1,4-galactose residues. The β1,4-galactose residues in EPO are important for *in vivo* hematopoietic activity [57], but their contribution toward cytoprotective function remains to be determined. Our results (**Figure 6A**) clearly show that asialo-rhuEPO^P^ with heterogeneous *N*-glycans possesses good cytoprotective activity, implying that β1,4-galactose residues may not be necessary for cytoprotective function. If future comparative studies reveal better cytoprotective effects for β1,4-galactose-extended asialo-rhuEPO^P^ than asialo-rhuEPO^P^ with heterogeneous *N*-glycans, we will improve the β1,4-galactosylation efficiency by knocking down hexosaminidases [56] or by introducing chimeric *GalT* (*STlGalT*) gene to express a chimeric enzyme containing the *N*-terminal sequences of rat sialyltransferase and catalytic domain of human GalT [58]. The presence of *N*-glycans with plant-specific β1,2-xylose and core α1,3-fucose on asialo-rhuEPO^P^ (**Figure 4** **;** **Table 1**) may also be of concern because of perceived immunogenicity of β1,2-xylose and α1,3-fucose residues, although the results of immunization with plant-produced glycoproteins in animal models are inconsistent to implicate these residues in causing adverse effects [59]–[61]. Recently, FDA approved intravenous injection of Elelyso^TM^, a carrot cell-produced human glucocerbrosidase bearing β1,2-xylose and core α1,3-fucose residues for long-term enzyme replacement therapy to treat type 1 Gaucher's disease because it did not display any adverse effects during clinical trials (http://www.fda.gov/NewsEvents/Newsroom/PressAnnouncements/ucm302549.htm). Nevertheless, for regulatory and safety issues, β1,2-xylose and α1,3-fucose residues on asialo-rhuEPO^P^ can be eliminated by knocking down genes encoding β1,2-xylosyltransferase and α1,3-fucosyltransferase in transgenic tobacco plants expressing asialo-rhuEPO, as done earlier by Strasser et al. [62] and Cox et al. [63].

The results obtained from the *in vitro* cytoprotection assay clearly show that asialo-rhuEPO^P^ can protect neuronal-like N2A cells from STS-induced cell death (**Figure 6A**). The cytoprotective effect of asialo-rhuEPO^P^ was superior to that observed with rhuEPO^M^. The heterogeneity of asialo-rhuEPO^P^ *N*-glycans seems not to affect its cytoprotective activity. The precise mechanism responsible for superior cytoprotective effect of the asialo-rhuEPO^P^ is not known yet, but it might be due to its increased association rate with EPOR than rhuEPO^M^, which leads to rapid activation of EPOR resulting in faster and stronger intracellular cell survival signal [64]. Concerning the molecular mechanisms by which rhuEPO and its derivatives provide cytoprotection, the majority of tissue protective responses is known to begin by the phosphorylation of JAK2 [3], followed by the activation of STAT5-Bcl-xL [65], PI3K/Akt [66] and MAPK [67] pathways. Both MAPK and PI3K/Akt inhibit caspase activation, thus directly mitigating apoptosis [68], [69]. Given these observations and the fact that asialo-rhuEPO^P^ treatment activated JAK2 and prevented caspase 3 activation (**Figure 6B**), we speculate that the asialo-rhuEPO^P^ protected N2A cells from STS-induced death by JAK2-mediated activation of MAPK or PI3K/Akt pathways leading to inhibition of caspase 3 activation, thus attenuating apoptosis.

In summary, we have demonstrated that the asialo-rhuEPO^P^ displays excellent cytoprotective activity by the phosphorylation of JAK2 and the inhibition of caspase 3 activation, thus decreasing apoptosis. Therefore, plant-based expression system lacking sialylating capacity represents a promising expression system for producing functionally active asialo-rhuEPO, which could be tested in broader tissue and cell protection areas.

## Supporting Information

Figure S1 **MS/MS spectra of tryptic peptides derived from 30 kD protein band of plant-produced asialo-rhuEPO (A-F)**. Each spectrum indicates the amino acid sequence of tryptic peptide whose position in human EPO amino acid sequence is shown in red (G).
(PDF)

Figure S2 **MS/MS spectra of tryptic peptides derived from 28 kD protein band of plant-produced asialo-rhuEPO (A-G).** Each spectrum indicates the amino acid sequence of tryptic peptide whose position in human EPO amino acid sequence is shown in red (H).
(PDF)

Figure S3 **MS/MS spectra of asialo-rhuEPO^P^** ***N*** **-glycans (A-M).** Total ion mapping was performed on PNGase A released and permethylated *N*-glycans to obtained MS/MS spectra.
(PDF)
