# Supplementary material for: Cytoprotective Effect of Recombinant Human Erythropoietin Produced in Transgenic Tobacco Plants
Source: PLoS One. 2013 Oct 4;8(10):e76468. doi: 10.1371/journal.pone.0076468 (PMC3790672; doi:10.1371/journal.pone.0076468)
Supplement: Figure S2 — MS/MS spectra of tryptic peptides derived from 28 kD protein band of plant-produced asialo-rhuEPO (A-G). Each spectrum indicates the amino acid sequence of tryptic peptide whose position in human EPO amino acid sequence is shown in red (H). (PDF) [file pone.0076468.s002.pdf]

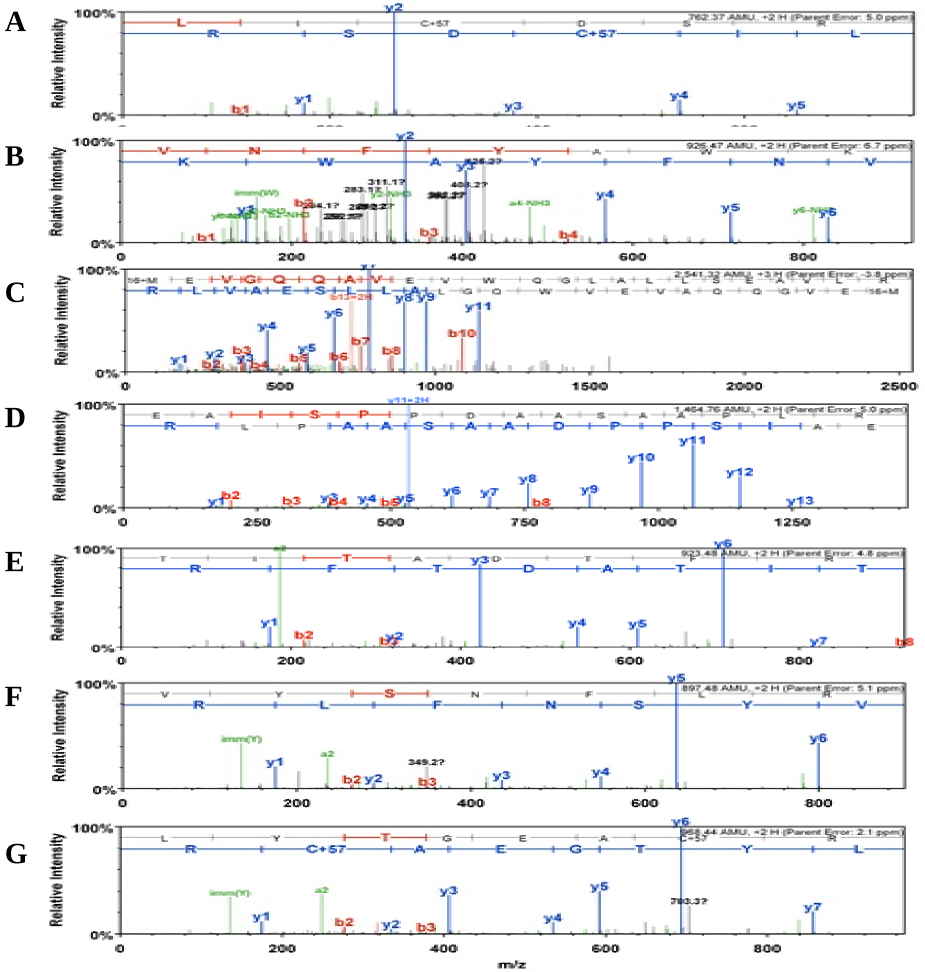

H

APPRLICDSRVLQRYLLEAKEAENITTGCAEHCSLNE
NITVPDTKVNFYAWKRMEVGQQAVEVWQGLALLS
EAVLRGQALLVNSSQPWEPLQLHVDKAVSGLRSLTT
LLRALGAQKEAISPPDAASAAPLRTITADTFRKLFRVY
SNFLRGKLKLYTGEACRTGDR

Figure S2. MS/MS spectra of tryptic peptides derived from 28 kD protein band of plant-produced asialo-rhuEPO (A-G). Each spectrum indicates the amino acid sequence of tryptic peptide whose position in human EPO amino acid sequence is shown in red (H).
