# Supplementary material for: Cytoprotective Effect of Recombinant Human Erythropoietin Produced in Transgenic Tobacco Plants
Source: PLoS One. 2013 Oct 4;8(10):e76468. doi: 10.1371/journal.pone.0076468 (PMC3790672; doi:10.1371/journal.pone.0076468)
Supplement: Figure S3 — MS/MS spectra of asialo-rhuEPOP N -glycans (A-M). Total ion mapping was performed on PNGase A released and permethylated N-glycans to obtained MS/MS spectra. (PDF) [file pone.0076468.s003.pdf]

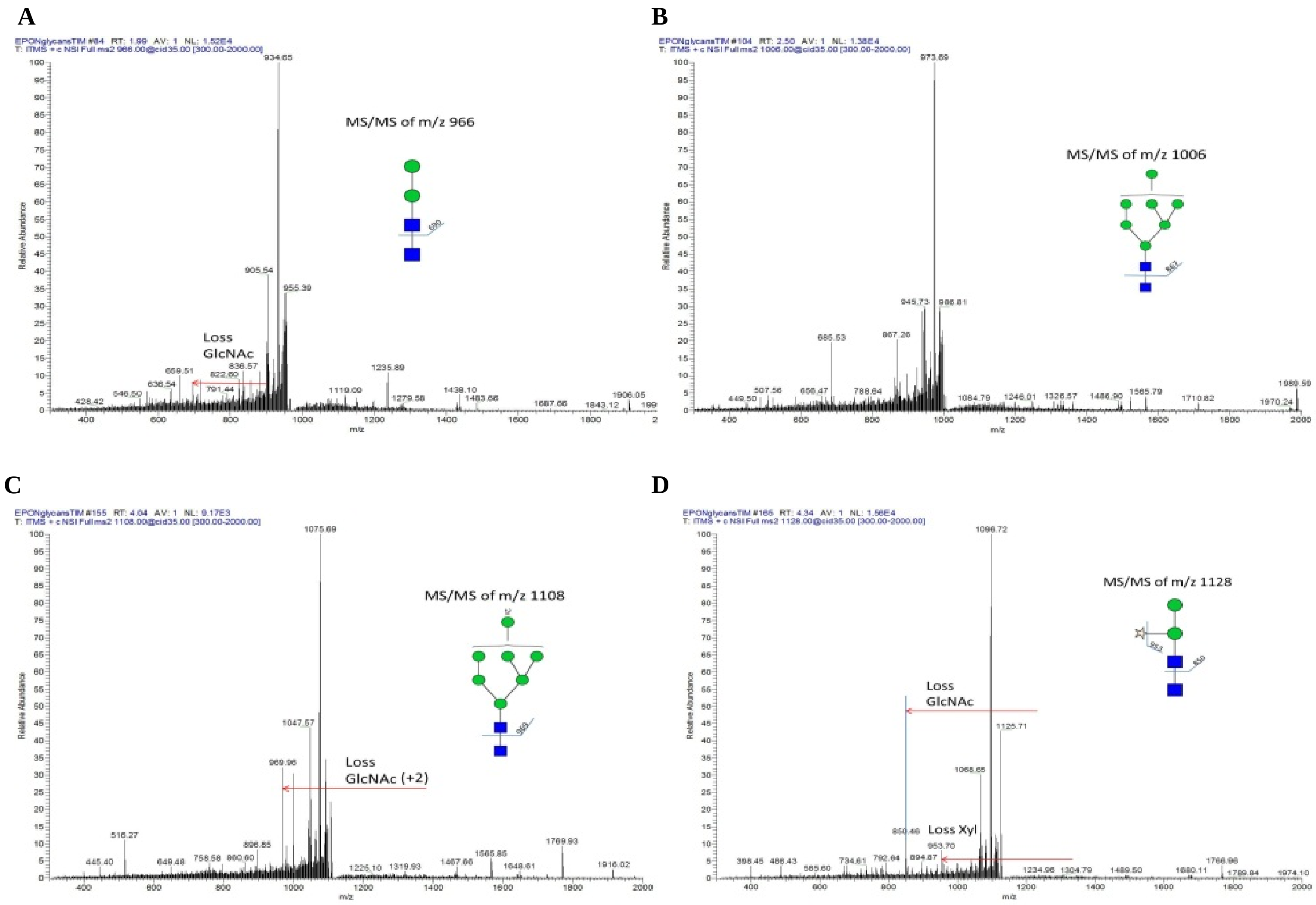
A
MS/MS of m/z 966
Loss
GlcNAc
B
MS/MS of m/z 1006
C
MS/MS of m/z 1108
Loss
GlcNAc (+2)
D
MS/MS of m/z 1128
Loss
GlcNAc
Loss Xyl

Figure S3A-D

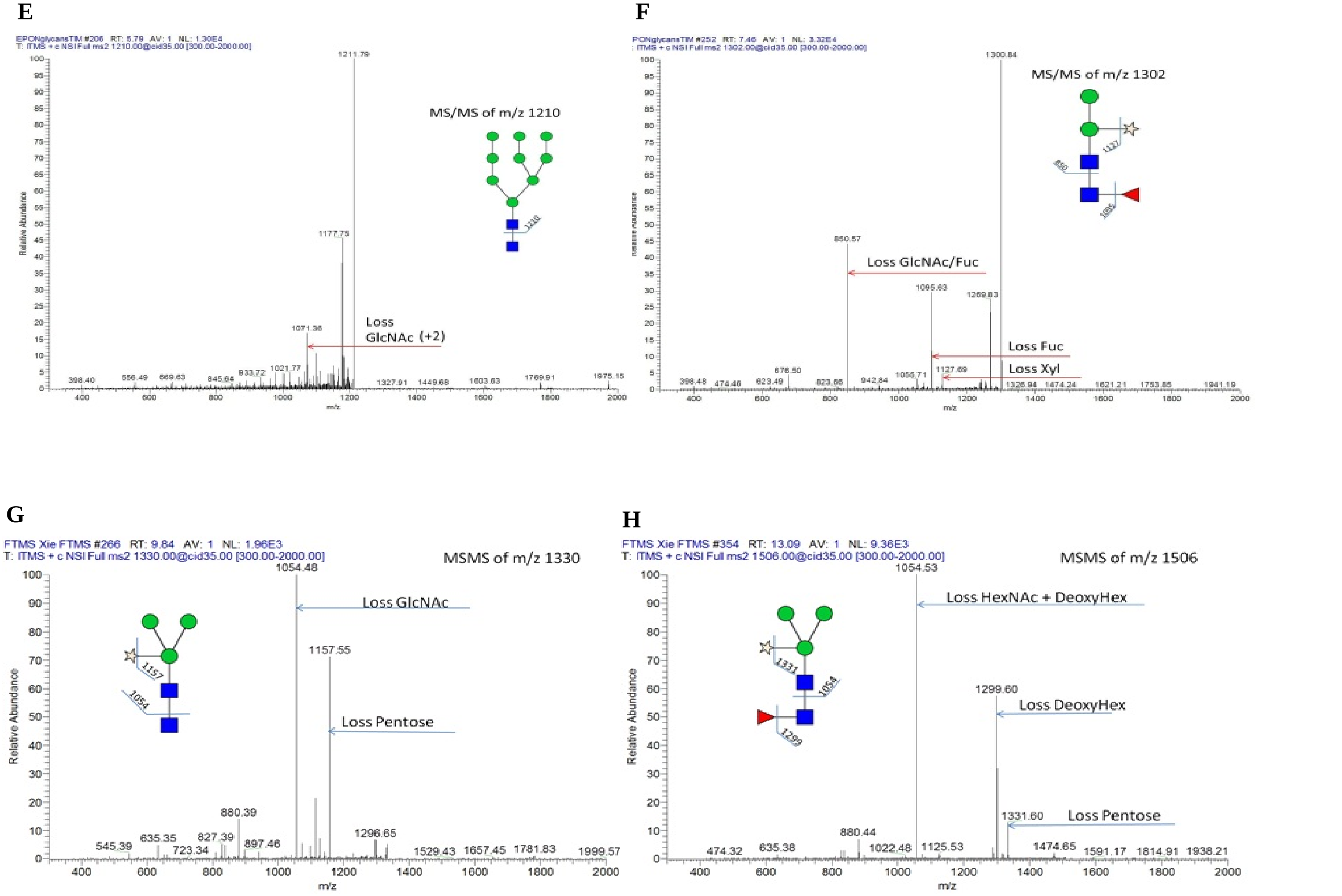
E
MS/MS of m/z 1210
Loss
GlcNAc (+2)
F
MS/MS of m/z 1302
Loss GlcNAc/Fuc
Loss Fuc
Loss Xyl
G
FTMS Xie FTMS #266 RT: 9.84 AV: 1 NL: 1.96E3
T: ITMS + c NSI Full ms2 1330.00@cid35.00 [300.00-2000.00]
MSMS of m/z 1330
Loss GlcNAc
Loss Pentose
Relative Abundance
m/z
H
FTMS Xie FTMS #354 RT: 13.09 AV: 1 NL: 9.36E3
T: ITMS + c NSI Full ms2 1506.00@cid35.00 [300.00-2000.00]
MSMS of m/z 1506
Loss HexNAc + DeoxyHex
Loss DeoxyHex
Loss Pentose
Relative Abundance
m/z

Figure S3E-H

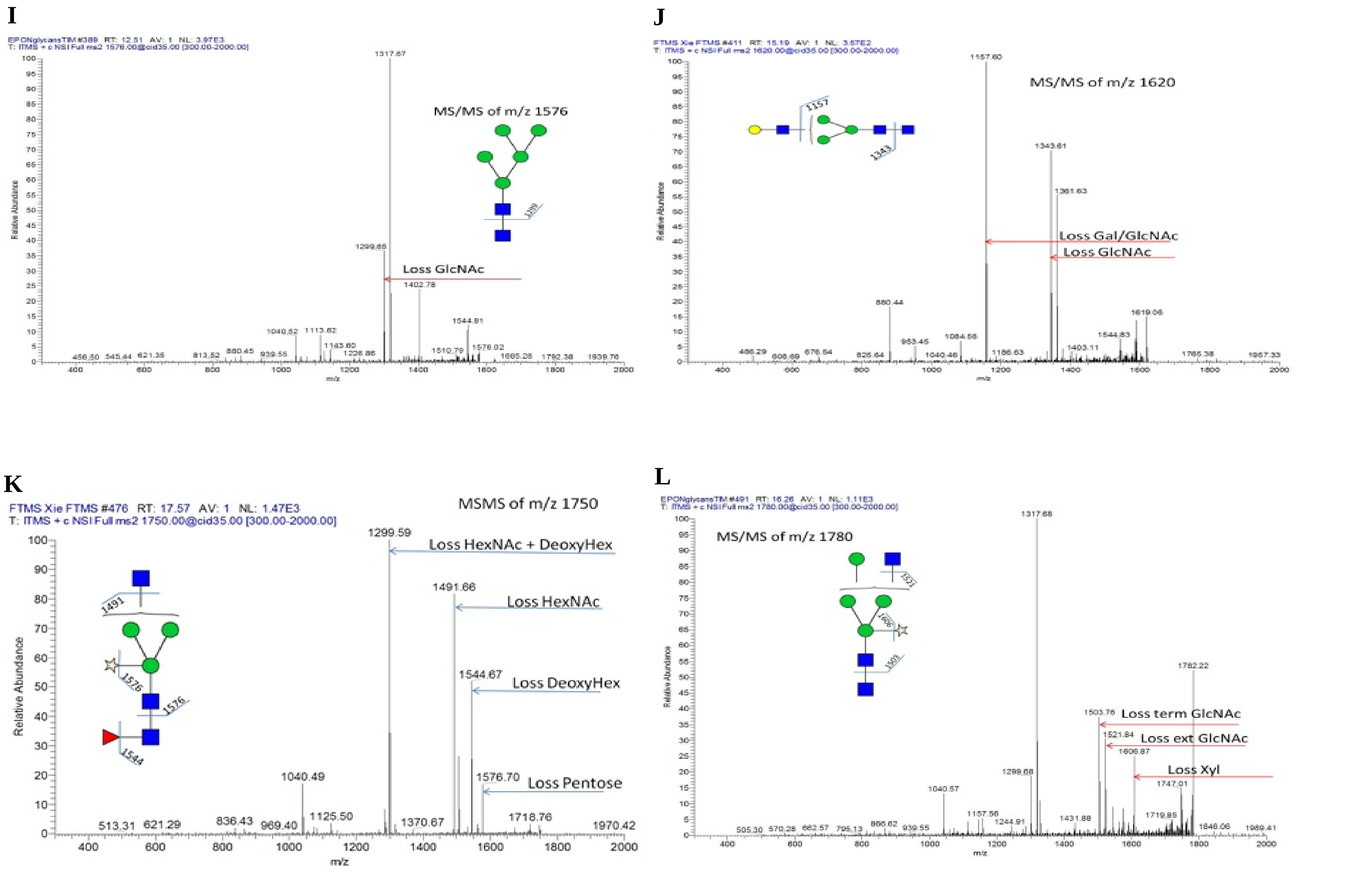
I
MS/MS of m/z 1576
Loss GlcNAc
1317.67
1299.65
1402.78
1544.81
J
MS/MS of m/z 1620
Loss Gal/GlcNAc
Loss GlcNAc
1157.60
1343.61
1361.63
880.44
1619.06
K
FTMS Xie FTMS #476 RT: 17.57 AV: 1 NL: 1.47E3
T: ITMS + c NSI Full ms2 1750.00@cid35.00 [300.00-2000.00]
MSMS of m/z 1750
Loss HexNAc + DeoxyHex
Loss HexNAc
Loss DeoxyHex
Loss Pentose
1299.59
1491.66
1544.67
1576.70
1040.49
1125.50
1370.67
1718.76
1970.42
513.31
621.29
836.43
969.40
Relative Abundance
m/z
L
MS/MS of m/z 1780
Loss term GlcNAc
Loss ext GlcNAc
Loss Xyl
1317.68
1782.22

Figure S3I-L

M

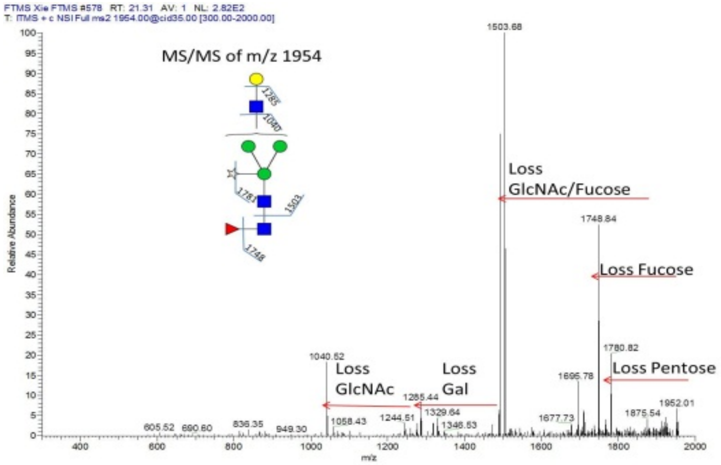

Figure S3. MS/MS spectra of asialo-rhuEPO[P] *N*-glycans (A-M). Total ion mapping was performed on PNGase A released and permethylated *N*-glycans to obtained MS/MS spectra.
